# Supplementary material for: Saccharomyces boulardii (CNCM I-745) improves intestinal damage in sepsis by remodeling intestinal flora structure
Source: Front Cell Infect Microbiol. 2025 Jul 31;15:1602792. doi: 10.3389/fcimb.2025.1602792 (PMC12350329; doi:10.3389/fcimb.2025.1602792)
Supplement: Supplementary file 1 [file DataSheet1.zip › summary/summary/7_Differential_analysis/1_diff_abundance/4_Order/Group.DW4_vs_DW0_vs_CW4_vs_CW0_vs_BW4_vs_BW0_vs_AW4_vs_AW0/Group.DW4_vs_DW0_vs_CW4_vs_CW0_vs_BW4_vs_BW0_vs_AW4_vs_AW0_diff_barplot.pdf]

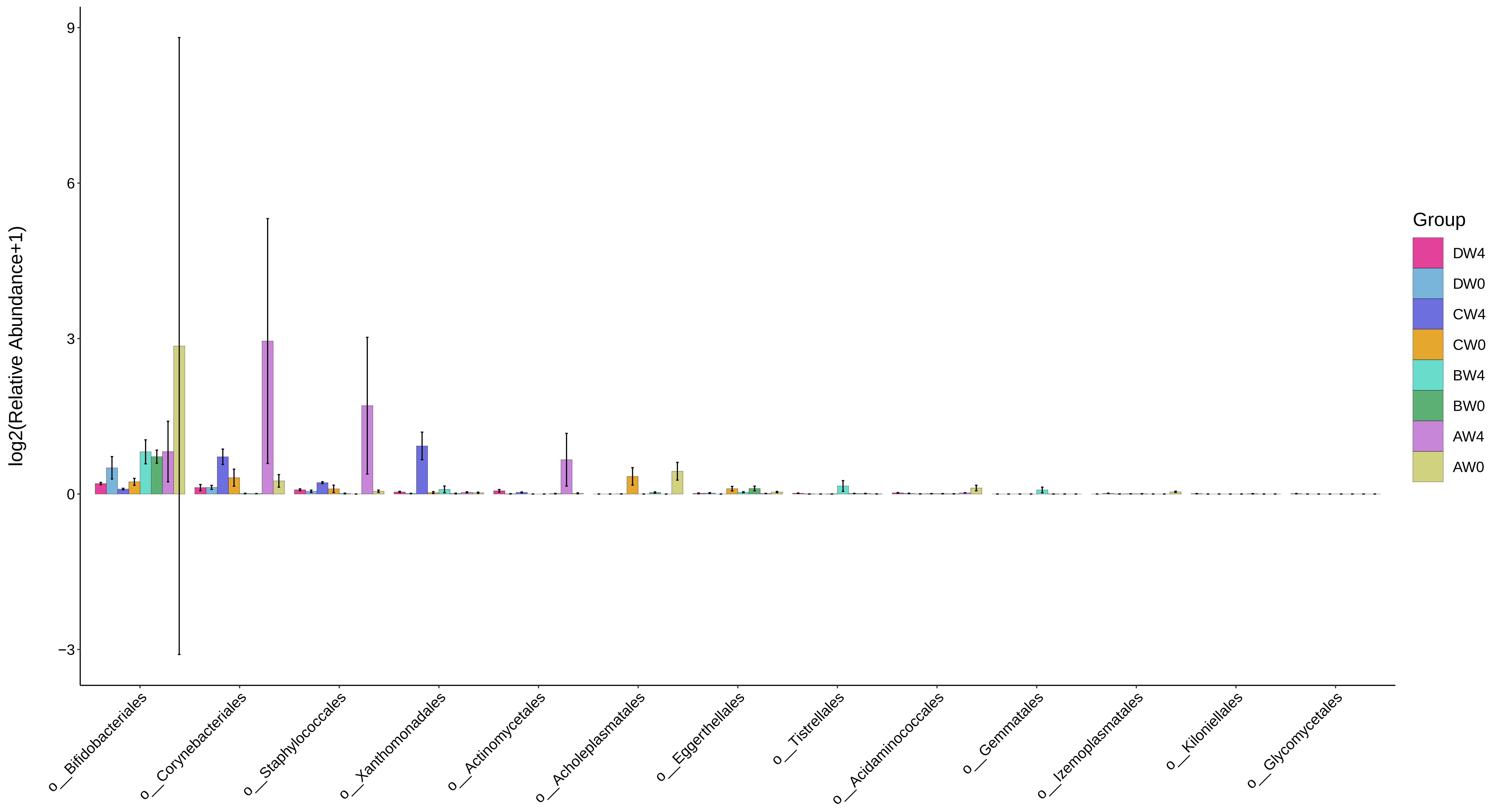

log2(Relative Abundance+1)
9
6
3
0
−3
o__Bifidobacteriales
o__Corynebacteriales
o__Staphylococcales
o__Xanthomonadales
o__Actinomycetales
o__Acholeplasmatales
o__Eggerthellales
o__Tistrellales
o__Acidaminococcales
o__Gemmatales
o__Izemoplasmatales
o__Kiloniellales
o__Glycomycetales
Group
DW4
DW0
CW4
CW0
BW4
BW0
AW4
AW0
